# 9-(2-Hy­droxy-4,4-dimethyl-6-oxocyclo­hex-1-en-1-yl)-3,3-dimethyl-2,3,4,9-tetra­hydro-1*H*-xanthen-1-one

**DOI:** 10.1107/S1600536812018934

**Published:** 2012-05-02

**Authors:** Malahat M. Kurbanova, Atash V. Gurbanov, Kamran T. Mahmudov, Abel M. Maharramov, Seik Weng Ng

**Affiliations:** a Department of Organic Chemistry, Baku State University, Baku, Azerbaijan; b Department of Chemistry, University of Malaya, 50603 Kuala Lumpur, Malaysia; c Chemistry Department, Faculty of Science, King Abdulaziz University, PO Box 80203 Jeddah, Saudi Arabia

## Abstract

The cyclo­hexene ring that constitutes a part of the tetra­hydroxanthene fused-ring system of the title compound, C_23_H_26_O_4_, adopts a flattened half-chair conformation that approximates an envelope conformation (in which the methyl­ene C atom bearing the two methyl substituents represents the flap) as five of the six atoms lie approximately on a plane (r.m.s. deviation = 0.020 Å). The mean plane of the cyclo­hexene ring with the hy­droxy substituent is approximately perpendicular to the mean plane of the tetra­hydroxanthene system. In the crystal, adjacent mol­ecules are linked by O—H⋯O_carbon­yl_ hydrogen bonds into a chain running along the *b* axis.

## Related literature

For the synthesis, see: Pyrko (1996 ▶).
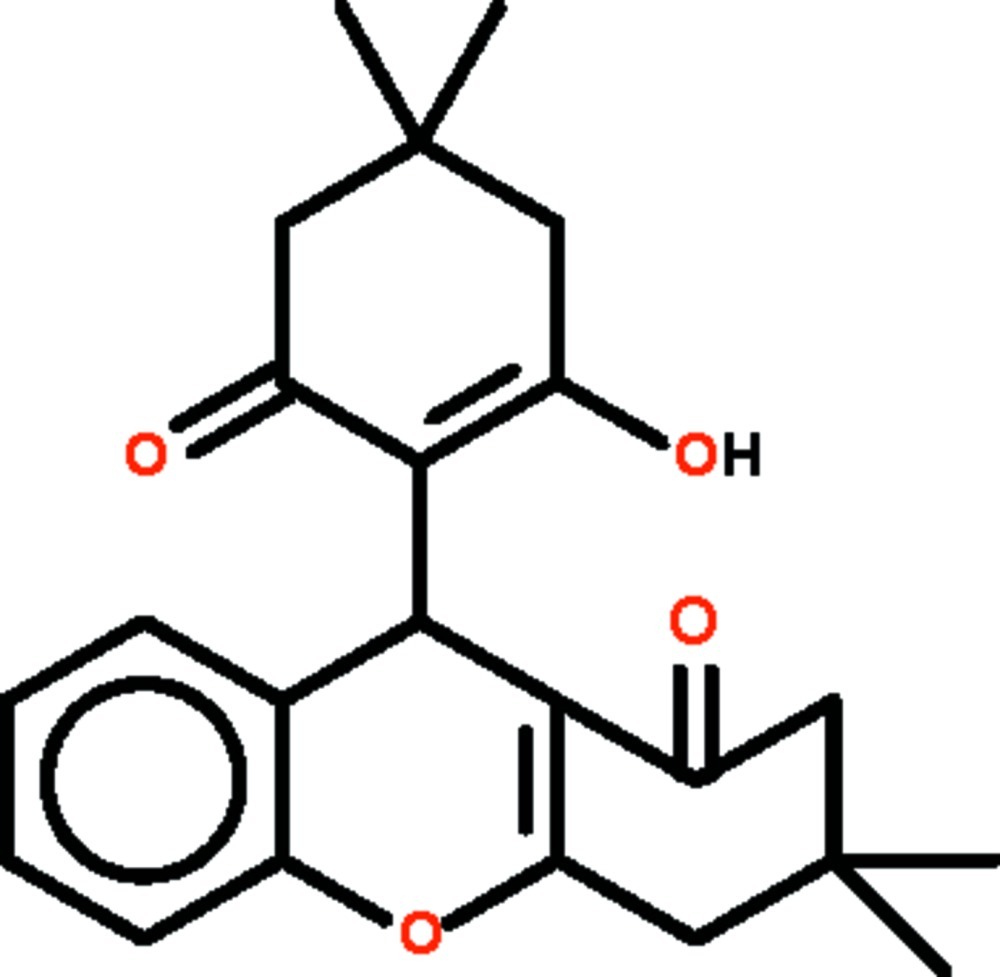


## Experimental

### 

#### Crystal data

C_23_H_26_O_4_
*M* *_r_* = 366.44
Orthorhombic, 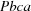

*a* = 15.3583 (5) Å
*b* = 11.3833 (4) Å
*c* = 22.2070 (7) Å
*V* = 3882.4 (2) Å^3^
*Z* = 8
Mo *K*α radiation
μ = 0.09 mm^−1^
*T* = 293 K
0.3 × 0.2 × 0.2 mm

#### Data collection

Bruker SMART APEX diffractometer
40229 measured reflections
4454 independent reflections
3311 reflections with *I* > 2σ(*I*)
*R*_int_ = 0.035

#### Refinement

*R*[*F* ^2^ > 2σ(*F* ^2^)] = 0.062
*wR*(*F* ^2^) = 0.195
*S* = 1.03
4454 reflections
251 parameters
2 restraints
H atoms treated by a mixture of independent and constrained refinement
Δρ_max_ = 0.87 e Å^−3^
Δρ_min_ = −0.28 e Å^−3^

### 

Data collection: *APEX2* (Bruker, 2005 ▶); cell refinement: *SAINT* (Bruker, 2005 ▶); data reduction: *SAINT*; program(s) used to solve structure: *SHELXS97* (Sheldrick, 2008 ▶); program(s) used to refine structure: *SHELXL97* (Sheldrick, 2008 ▶); molecular graphics: *X-SEED* (Barbour, 2001 ▶); software used to prepare material for publication: *publCIF* (Westrip, 2010 ▶).

## Supplementary Material

Crystal structure: contains datablock(s) global, I. DOI: 10.1107/S1600536812018934/zs2203sup1.cif

Structure factors: contains datablock(s) I. DOI: 10.1107/S1600536812018934/zs2203Isup2.hkl

Supplementary material file. DOI: 10.1107/S1600536812018934/zs2203Isup3.cml

Additional supplementary materials:  crystallographic information; 3D view; checkCIF report

## Figures and Tables

**Table 1:** Hydrogen-bond geometry (Å, °)

*D*—H⋯*A*	*D*—H	H⋯*A*	*D*⋯*A*	*D*—H⋯*A*
O3—H1⋯O2^i^	0.83 (3)	1.90 (3)	2.706 (2)	165 (3)

Symmetry code: (i) 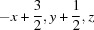.

**Fig. 1.**
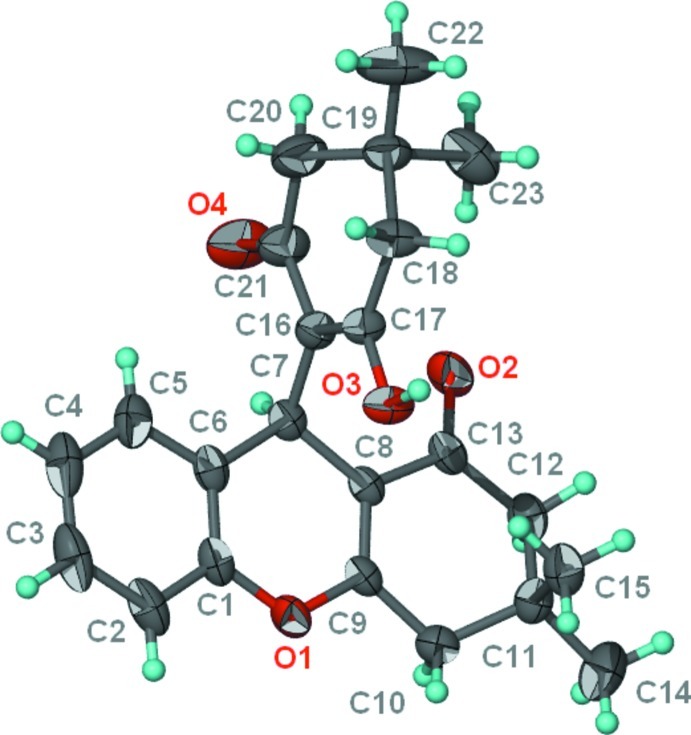
Thermal ellipsoid plot (Barbour, 2001) of C23H26O4 at the 50% probability level. Hydrogen atoms are drawn as spheres of arbitrary radius. The disorder is not shown.

**Fig. 2.**
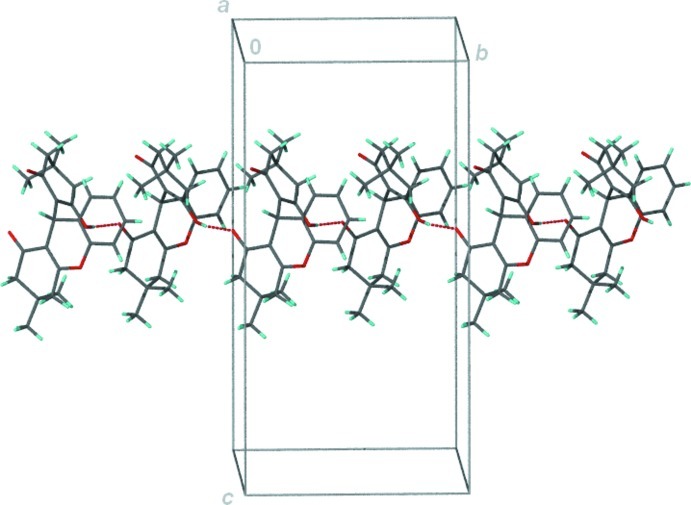
The hydrogen-bonded chain motif showing hydrogen bonds as dashed lines..

## supplementary crystallographic information

### Comment

Dimedone condenses with an aromatic aldehyde such an salicylaldehyde to yield aldimedone, the reaction often being used as a method for characterizing aromatic aldehydes. Aldimedone is then dehydrated to the title compound C_23_H_26_O_4_ (Scheme I), which features a pyran ring (Pyrko, 1996). The cyclohexene ring that constitutes a part of the tetrahydroxanthene fused-ring system has a flattened half-chair conformation that approximates an envelope conformation (in which the methylene C atom bearing the dimethyl substituent represents the flap) as five of the six atoms lie on a plane (Fig. 1). The mean plane of the cyclohexene ring with the hydroxy substituent is approximately perpendicular to the mean plane of the tetrahydroxanthene system. Adjacent molecules are linked by an *O*–*H*···O_carbonyl_ hydrogen bond to form a chain (Fig. 2, Table 1), running along the *b*-axis of the orthorhombic unit cell.

### Experimental

The compound was synthesized by using a literature method (Pyrko, 1996) and ethanol was used as the recrystallization solvent.

### Refinement

Carbon-bound H-atoms were placed in calculated positions [C—H = 0.93–0.98 Å; *U*_iso_(H) = 1.2 or 1.5*U*_eq_(C)] and were included in the refinement in the riding model approximation. The hydroxy H-atom was located in a difference Fourier map and was freely refined. One methylene C atom is disordered over two positions with the disorder assumed to be a 1:1 type. 1,2-Related bond distances involving the disordered atoms were restrained to within 0.01 Å of each other and the temperature factors were restrained to be equal. The atoms are separated by 0.25 Å.

### Crystal data

**Table:**

C_23_H_26_O_4_	*F*(000) = 1568
*M**_r_* = 366.44	*D*_x_ = 1.254 Mg m^−^^3^
Orthorhombic, *P**b**c**a*	Mo *K*α radiation, λ = 0.71073 Å
Hall symbol: -P 2ac 2ab	Cell parameters from 7520 reflections
*a* = 15.3583 (5) Å	θ = 2.3–26.4°
*b* = 11.3833 (4) Å	µ = 0.09 mm^−^^1^
*c* = 22.2070 (7) Å	*T* = 293 K
*V* = 3882.4 (2) Å^3^	Prism, colorless
*Z* = 8	0.3 × 0.2 × 0.2 mm

### Data collection

**Table:**

Bruker SMART APEX diffractometer	3311 reflections with *I* > 2σ(*I*)
Radiation source: fine-focus sealed tube	*R*_int_ = 0.035
Graphite monochromator	θ_max_ = 27.5°, θ_min_ = 1.8°
φ and ω scans	*h* = −19→19
40229 measured reflections	*k* = −14→14
4454 independent reflections	*l* = −28→28

### Refinement

**Table:**

Refinement on *F*^2^	Primary atom site location: structure-invariant direct methods
Least-squares matrix: full	Secondary atom site location: difference Fourier map
*R*[*F*^2^ > 2σ(*F*^2^)] = 0.062	Hydrogen site location: inferred from neighbouring sites
*wR*(*F*^2^) = 0.195	H atoms treated by a mixture of independent and constrained refinement
*S* = 1.03	*w* = 1/[σ^2^(*F*_o_^2^) + (0.0987*P*)^2^ + 2.2761*P*] where *P* = (*F*_o_^2^ + 2*F*_c_^2^)/3
4454 reflections	(Δ/σ)_max_ = 0.001
251 parameters	Δρ_max_ = 0.87 e Å^−^^3^
2 restraints	Δρ_min_ = −0.28 e Å^−^^3^

### Fractional atomic coordinates and isotropic or equivalent isotropic displacement parameters (Å^2^)

**Table:**

	*x*	*y*	*z*	*U*_iso_*/*U*_eq_	Occ. (<1)
O1	0.48195 (10)	0.28998 (14)	0.52034 (7)	0.0505 (4)	
O2	0.66264 (11)	−0.01741 (14)	0.45598 (8)	0.0554 (4)	
O3	0.70445 (11)	0.32716 (13)	0.44902 (7)	0.0452 (4)	
O4	0.59639 (14)	0.0636 (2)	0.30630 (10)	0.0834 (7)	
C1	0.46916 (14)	0.33493 (18)	0.46269 (11)	0.0434 (5)	
C2	0.41821 (15)	0.4357 (2)	0.45996 (14)	0.0595 (7)	
H2	0.3945	0.4680	0.4948	0.071*	
C3	0.40360 (17)	0.4863 (2)	0.40516 (16)	0.0677 (8)	
H3	0.3701	0.5542	0.4027	0.081*	
C4	0.43765 (17)	0.4385 (2)	0.35362 (15)	0.0675 (8)	
H4	0.4267	0.4731	0.3165	0.081*	
C5	0.48839 (16)	0.3382 (2)	0.35732 (12)	0.0554 (6)	
H5	0.5113	0.3057	0.3223	0.067*	
C6	0.50576 (13)	0.28513 (18)	0.41254 (10)	0.0410 (5)	
C7	0.56366 (13)	0.17701 (16)	0.41671 (9)	0.0366 (4)	
H7	0.5317	0.1125	0.3976	0.044*	
C8	0.57658 (12)	0.14286 (16)	0.48175 (9)	0.0341 (4)	
C9	0.53658 (13)	0.19768 (17)	0.52763 (9)	0.0377 (4)	
C10	0.54466 (15)	0.1665 (2)	0.59216 (10)	0.0470 (5)	
H10A	0.4980	0.1130	0.6029	0.056*	
H10B	0.5381	0.2370	0.6163	0.056*	
C11	0.63217 (15)	0.1089 (2)	0.60688 (10)	0.0468 (5)	
C12	0.64636 (16)	0.01043 (19)	0.56117 (11)	0.0483 (5)	
H12A	0.7057	−0.0178	0.5653	0.058*	
H12B	0.6079	−0.0542	0.5713	0.058*	
C13	0.63134 (13)	0.04274 (17)	0.49627 (10)	0.0390 (5)	
C14	0.6290 (2)	0.0584 (3)	0.67047 (13)	0.0754 (9)	
H14A	0.6211	0.1210	0.6989	0.113*	
H14B	0.6826	0.0182	0.6789	0.113*	
H14C	0.5813	0.0042	0.6736	0.113*	
C15	0.70536 (16)	0.1997 (2)	0.60273 (12)	0.0550 (6)	
H15A	0.6957	0.2606	0.6319	0.083*	
H15B	0.7062	0.2332	0.5631	0.083*	
H15C	0.7602	0.1622	0.6106	0.083*	
C16	0.64720 (13)	0.19297 (18)	0.38117 (9)	0.0389 (4)	
C17	0.71267 (13)	0.26420 (16)	0.39816 (9)	0.0372 (4)	
C18	0.79608 (16)	0.2789 (2)	0.36444 (11)	0.0516 (6)	
H18A	0.8438	0.2783	0.3930	0.062*	
H18B	0.7958	0.3552	0.3450	0.062*	
C19	0.81293 (16)	0.1858 (2)	0.31721 (11)	0.0500 (6)	
C20	0.7277 (6)	0.168 (3)	0.2842 (3)	0.066 (3)	0.50
H20A	0.7120	0.2404	0.2643	0.079*	0.50
H20B	0.7362	0.1085	0.2533	0.079*	0.50
C20'	0.7318 (6)	0.146 (3)	0.2843 (3)	0.066 (3)	0.50
H20C	0.7179	0.2033	0.2534	0.079*	0.50
H20D	0.7440	0.0721	0.2643	0.079*	0.50
C21	0.65304 (17)	0.1301 (2)	0.32425 (11)	0.0572 (6)	
C22	0.8863 (2)	0.2210 (4)	0.27608 (16)	0.0985 (13)	
H22A	0.8698	0.2895	0.2536	0.148*	
H22B	0.8988	0.1578	0.2488	0.148*	
H22C	0.9372	0.2381	0.2996	0.148*	
C23	0.8426 (2)	0.0735 (3)	0.35122 (17)	0.0787 (9)	
H23A	0.7973	0.0485	0.3781	0.118*	
H23B	0.8944	0.0904	0.3739	0.118*	
H23C	0.8546	0.0122	0.3227	0.118*	
H1	0.751 (2)	0.364 (3)	0.4533 (13)	0.075 (9)*	

### Atomic displacement parameters (Å^2^)

**Table:**

	*U*^11^	*U*^22^	*U*^33^	*U*^12^	*U*^13^	*U*^23^
O1	0.0498 (9)	0.0477 (9)	0.0541 (9)	0.0205 (7)	−0.0029 (7)	−0.0078 (7)
O2	0.0590 (10)	0.0420 (8)	0.0652 (10)	0.0186 (7)	0.0003 (8)	−0.0105 (8)
O3	0.0470 (9)	0.0411 (8)	0.0475 (9)	−0.0143 (7)	0.0084 (7)	−0.0158 (7)
O4	0.0751 (13)	0.1020 (16)	0.0732 (13)	−0.0333 (12)	0.0031 (10)	−0.0475 (12)
C1	0.0348 (10)	0.0341 (10)	0.0612 (13)	0.0025 (8)	−0.0118 (9)	−0.0023 (9)
C2	0.0423 (12)	0.0409 (12)	0.095 (2)	0.0093 (10)	−0.0140 (12)	−0.0044 (12)
C3	0.0469 (14)	0.0409 (12)	0.115 (3)	0.0025 (10)	−0.0264 (15)	0.0163 (15)
C4	0.0495 (14)	0.0624 (16)	0.091 (2)	−0.0052 (12)	−0.0231 (14)	0.0318 (16)
C5	0.0455 (12)	0.0605 (15)	0.0602 (15)	−0.0054 (11)	−0.0149 (11)	0.0113 (12)
C6	0.0310 (9)	0.0352 (10)	0.0569 (13)	−0.0040 (8)	−0.0105 (9)	0.0012 (9)
C7	0.0332 (9)	0.0316 (9)	0.0451 (11)	−0.0046 (8)	−0.0049 (8)	−0.0059 (8)
C8	0.0279 (9)	0.0280 (9)	0.0465 (11)	0.0000 (7)	−0.0009 (7)	−0.0011 (8)
C9	0.0296 (9)	0.0348 (10)	0.0489 (11)	0.0031 (7)	−0.0011 (8)	−0.0025 (8)
C10	0.0452 (12)	0.0503 (13)	0.0455 (12)	0.0032 (9)	0.0048 (9)	−0.0025 (10)
C11	0.0504 (12)	0.0445 (11)	0.0455 (12)	0.0017 (10)	−0.0058 (9)	0.0056 (9)
C12	0.0483 (12)	0.0356 (10)	0.0609 (14)	0.0041 (9)	−0.0060 (10)	0.0069 (10)
C13	0.0327 (9)	0.0296 (9)	0.0547 (12)	0.0007 (8)	−0.0024 (8)	−0.0024 (8)
C14	0.091 (2)	0.0772 (19)	0.0584 (16)	−0.0007 (17)	−0.0129 (15)	0.0207 (14)
C15	0.0500 (13)	0.0497 (13)	0.0653 (15)	0.0015 (10)	−0.0146 (11)	−0.0025 (11)
C16	0.0389 (10)	0.0386 (10)	0.0393 (10)	−0.0028 (8)	−0.0005 (8)	−0.0069 (8)
C17	0.0431 (10)	0.0304 (9)	0.0380 (10)	−0.0038 (8)	0.0038 (8)	−0.0055 (8)
C18	0.0533 (13)	0.0459 (12)	0.0555 (13)	−0.0143 (10)	0.0165 (11)	−0.0131 (10)
C19	0.0489 (12)	0.0559 (13)	0.0451 (12)	−0.0021 (10)	0.0076 (10)	−0.0149 (10)
C20	0.072 (2)	0.078 (9)	0.0491 (14)	−0.002 (3)	0.0043 (13)	−0.0216 (17)
C20'	0.072 (2)	0.078 (9)	0.0491 (14)	−0.002 (3)	0.0043 (13)	−0.0216 (17)
C21	0.0563 (14)	0.0649 (15)	0.0504 (13)	−0.0099 (12)	0.0013 (11)	−0.0213 (12)
C22	0.098 (2)	0.114 (3)	0.084 (2)	−0.041 (2)	0.051 (2)	−0.044 (2)
C23	0.0643 (17)	0.0632 (17)	0.108 (3)	0.0111 (14)	0.0051 (17)	−0.0101 (17)

### Geometric parameters (Å, º)

**Table:**

O1—C9	1.354 (2)	C12—H12A	0.9700
O1—C1	1.393 (3)	C12—H12B	0.9700
O2—C13	1.225 (3)	C14—H14A	0.9600
O3—C17	1.344 (2)	C14—H14B	0.9600
O3—H1	0.83 (3)	C14—H14C	0.9600
O4—C21	1.220 (3)	C15—H15A	0.9600
C1—C6	1.370 (3)	C15—H15B	0.9600
C1—C2	1.390 (3)	C15—H15C	0.9600
C2—C3	1.365 (4)	C16—C17	1.346 (3)
C2—H2	0.9300	C16—C21	1.456 (3)
C3—C4	1.371 (4)	C17—C18	1.493 (3)
C3—H3	0.9300	C18—C19	1.513 (3)
C4—C5	1.384 (4)	C18—H18A	0.9700
C4—H4	0.9300	C18—H18B	0.9700
C5—C6	1.393 (3)	C19—C22	1.505 (4)
C5—H5	0.9300	C19—C20'	1.514 (7)
C6—C7	1.521 (3)	C19—C20	1.514 (7)
C7—C8	1.509 (3)	C19—C23	1.553 (4)
C7—C16	1.517 (3)	C20—C21	1.513 (7)
C7—H7	0.9800	C20—H20A	0.9700
C8—C9	1.344 (3)	C20—H20B	0.9700
C8—C13	1.453 (3)	C20'—C21	1.510 (7)
C9—C10	1.482 (3)	C20'—H20C	0.9700
C10—C11	1.530 (3)	C20'—H20D	0.9700
C10—H10A	0.9700	C22—H22A	0.9600
C10—H10B	0.9700	C22—H22B	0.9600
C11—C14	1.526 (3)	C22—H22C	0.9600
C11—C12	1.528 (3)	C23—H23A	0.9600
C11—C15	1.530 (3)	C23—H23B	0.9600
C12—C13	1.505 (3)	C23—H23C	0.9600
			
C9—O1—C1	118.84 (17)	H14B—C14—H14C	109.5
C17—O3—H1	107 (2)	C11—C15—H15A	109.5
C6—C1—C2	122.5 (2)	C11—C15—H15B	109.5
C6—C1—O1	122.50 (18)	H15A—C15—H15B	109.5
C2—C1—O1	115.0 (2)	C11—C15—H15C	109.5
C3—C2—C1	118.7 (3)	H15A—C15—H15C	109.5
C3—C2—H2	120.7	H15B—C15—H15C	109.5
C1—C2—H2	120.7	C17—C16—C21	119.59 (19)
C2—C3—C4	120.9 (2)	C17—C16—C7	123.94 (18)
C2—C3—H3	119.5	C21—C16—C7	116.41 (18)
C4—C3—H3	119.5	O3—C17—C16	119.13 (18)
C3—C4—C5	119.5 (3)	O3—C17—C18	116.26 (17)
C3—C4—H4	120.2	C16—C17—C18	124.61 (18)
C5—C4—H4	120.2	C17—C18—C19	114.57 (18)
C4—C5—C6	121.2 (3)	C17—C18—H18A	108.6
C4—C5—H5	119.4	C19—C18—H18A	108.6
C6—C5—H5	119.4	C17—C18—H18B	108.6
C1—C6—C5	117.2 (2)	C19—C18—H18B	108.6
C1—C6—C7	121.67 (19)	H18A—C18—H18B	107.6
C5—C6—C7	121.1 (2)	C22—C19—C18	111.2 (2)
C8—C7—C16	114.67 (16)	C22—C19—C20'	113.8 (3)
C8—C7—C6	110.10 (16)	C18—C19—C20'	113.8 (10)
C16—C7—C6	111.46 (17)	C22—C19—C20	113.0 (4)
C8—C7—H7	106.7	C18—C19—C20	106.4 (10)
C16—C7—H7	106.7	C22—C19—C23	107.1 (3)
C6—C7—H7	106.7	C18—C19—C23	106.8 (2)
C9—C8—C13	117.44 (19)	C20'—C19—C23	103.3 (12)
C9—C8—C7	123.10 (17)	C20—C19—C23	112.2 (12)
C13—C8—C7	119.39 (17)	C21—C20—C19	114.2 (6)
C8—C9—O1	123.57 (19)	C21—C20—H20A	108.7
C8—C9—C10	125.72 (18)	C19—C20—H20A	108.7
O1—C9—C10	110.71 (17)	C21—C20—H20B	108.7
C9—C10—C11	112.51 (18)	C19—C20—H20B	108.7
C9—C10—H10A	109.1	H20A—C20—H20B	107.6
C11—C10—H10A	109.1	C21—C20'—C19	114.3 (6)
C9—C10—H10B	109.1	C21—C20'—H20C	108.7
C11—C10—H10B	109.1	C19—C20'—H20C	108.7
H10A—C10—H10B	107.8	C21—C20'—H20D	108.7
C14—C11—C12	110.0 (2)	C19—C20'—H20D	108.7
C14—C11—C15	109.5 (2)	H20C—C20'—H20D	107.6
C12—C11—C15	110.5 (2)	O4—C21—C16	123.0 (2)
C14—C11—C10	109.3 (2)	O4—C21—C20'	117.0 (7)
C12—C11—C10	107.30 (18)	C16—C21—C20'	120.0 (7)
C15—C11—C10	110.09 (19)	O4—C21—C20	121.6 (6)
C13—C12—C11	115.78 (18)	C16—C21—C20	114.7 (8)
C13—C12—H12A	108.3	C19—C22—H22A	109.5
C11—C12—H12A	108.3	C19—C22—H22B	109.5
C13—C12—H12B	108.3	H22A—C22—H22B	109.5
C11—C12—H12B	108.3	C19—C22—H22C	109.5
H12A—C12—H12B	107.4	H22A—C22—H22C	109.5
O2—C13—C8	120.2 (2)	H22B—C22—H22C	109.5
O2—C13—C12	120.17 (19)	C19—C23—H23A	109.5
C8—C13—C12	119.54 (18)	C19—C23—H23B	109.5
C11—C14—H14A	109.5	H23A—C23—H23B	109.5
C11—C14—H14B	109.5	C19—C23—H23C	109.5
H14A—C14—H14B	109.5	H23A—C23—H23C	109.5
C11—C14—H14C	109.5	H23B—C23—H23C	109.5
H14A—C14—H14C	109.5		
			
C9—O1—C1—C6	3.9 (3)	C7—C8—C13—C12	178.33 (18)
C9—O1—C1—C2	−174.64 (19)	C11—C12—C13—O2	160.3 (2)
C6—C1—C2—C3	0.5 (3)	C11—C12—C13—C8	−22.2 (3)
O1—C1—C2—C3	179.0 (2)	C8—C7—C16—C17	−53.9 (3)
C1—C2—C3—C4	0.6 (4)	C6—C7—C16—C17	72.1 (3)
C2—C3—C4—C5	−0.8 (4)	C8—C7—C16—C21	128.9 (2)
C3—C4—C5—C6	−0.2 (4)	C6—C7—C16—C21	−105.2 (2)
C2—C1—C6—C5	−1.4 (3)	C21—C16—C17—O3	176.1 (2)
O1—C1—C6—C5	−179.84 (19)	C7—C16—C17—O3	−1.0 (3)
C2—C1—C6—C7	178.05 (19)	C21—C16—C17—C18	−4.3 (3)
O1—C1—C6—C7	−0.4 (3)	C7—C16—C17—C18	178.6 (2)
C4—C5—C6—C1	1.2 (3)	O3—C17—C18—C19	165.1 (2)
C4—C5—C6—C7	−178.2 (2)	C16—C17—C18—C19	−14.5 (3)
C1—C6—C7—C8	−3.4 (3)	C17—C18—C19—C22	167.0 (3)
C5—C6—C7—C8	176.07 (18)	C17—C18—C19—C20'	36.9 (10)
C1—C6—C7—C16	−131.8 (2)	C17—C18—C19—C20	43.6 (9)
C5—C6—C7—C16	47.6 (2)	C17—C18—C19—C23	−76.4 (3)
C16—C7—C8—C9	130.8 (2)	C22—C19—C20—C21	178.9 (14)
C6—C7—C8—C9	4.2 (3)	C18—C19—C20—C21	−59 (2)
C16—C7—C8—C13	−52.2 (2)	C20'—C19—C20—C21	82 (3)
C6—C7—C8—C13	−178.86 (16)	C23—C19—C20—C21	58 (2)
C13—C8—C9—O1	−178.15 (18)	C22—C19—C20'—C21	−170.2 (14)
C7—C8—C9—O1	−1.1 (3)	C18—C19—C20'—C21	−41 (2)
C13—C8—C9—C10	1.3 (3)	C20—C19—C20'—C21	−83 (3)
C7—C8—C9—C10	178.28 (19)	C23—C19—C20'—C21	74 (2)
C1—O1—C9—C8	−3.2 (3)	C17—C16—C21—O4	179.5 (3)
C1—O1—C9—C10	177.35 (18)	C7—C16—C21—O4	−3.1 (4)
C8—C9—C10—C11	28.1 (3)	C17—C16—C21—C20'	−0.7 (13)
O1—C9—C10—C11	−152.46 (19)	C7—C16—C21—C20'	176.7 (13)
C9—C10—C11—C14	−169.2 (2)	C17—C16—C21—C20	−9.8 (12)
C9—C10—C11—C12	−49.9 (2)	C7—C16—C21—C20	167.6 (12)
C9—C10—C11—C15	70.5 (3)	C19—C20'—C21—O4	−156.2 (14)
C14—C11—C12—C13	167.1 (2)	C19—C20'—C21—C16	24 (3)
C15—C11—C12—C13	−71.9 (2)	C19—C20'—C21—C20	83 (3)
C10—C11—C12—C13	48.2 (3)	C19—C20—C21—O4	−146.0 (13)
C9—C8—C13—O2	173.0 (2)	C19—C20—C21—C16	43 (2)
C7—C8—C13—O2	−4.2 (3)	C19—C20—C21—C20'	−82 (3)
C9—C8—C13—C12	−4.5 (3)		

### Hydrogen-bond geometry (Å, º)

**Table:**

*D*—H···*A*	*D*—H	H···*A*	*D*···*A*	*D*—H···*A*
O3—H1···O2^i^	0.83 (3)	1.90 (3)	2.706 (2)	165 (3)

Symmetry code: (i) −*x*+3/2, *y*+1/2, *z*.
